# Editorial: Omics driven research for the improvement of industrial crops

**DOI:** 10.3389/fpls.2023.1143571

**Published:** 2023-02-15

**Authors:** Rajarshi Kumar Gaur, Dinesh Yadav, Benedicte Riber Albrectsen

**Affiliations:** ^1^ Department of Biotechnology, Deen Dayal Upadhyaya Gorakhpur University, Gorakhpur, India; ^2^ Department of Plant Physiology, Umeå University, Umeå Plant Science Centre, Umeå, Sweden

**Keywords:** omics technology, industrial cash crops, long non-coding RNAs, stress, genetic variation

Over the years, we have witnessed several technological innovations in agriculture that assist breeders to enhance the productivity and yield of crops. These innovations are vital for achieving the goals set for global food and nutritional security, and they have already proven useful by improving crop resistance against biotic and abiotic stress factors.

With a plethora of sequenced plant genomes, omics-driven technologies are gaining importance over traditional plant breeding approaches. They provide a faster and more direct route to creating genetically improved crops, as they provide direct information about molecular processes in cells, tissues, and organs. Metabolomic, genomic, and transcriptomic techniques already help us identify genes of agronomic importance. Omics techniques further allow us to better understand the priorities and barriers that shape plant metabolism, and by using state-of-the-art omics tools, breeders may get valuable information about the organisation of the complete DNA in any crop of interest and learn about how this organisation is interpreted under various circumstances.

“*Omics-Driven Research for the Improvement of Industrial Crops*” is a collection of four papers that uses omics approaches to understand diverse features of plant metabolism during growth, in response to the surrounding environment, and in the crop afterlife. The papers range from trees to flax and cucumber, demonstrating the breadth of questions that can be answered using omics techniques.

The metabolic effects of below- and above-ground interactions for oak seedlings were studied by van Dijk et al. in a natural setting, i.e., with genetic variation, various assailants, and natural soil microbiomes. Although the studied soil microbiome did not seem to have a strong impact on the leaf metabolome, this study suggests that the metabolomic signature of oaks may be pushed in different directions depending on the antagonistic player; in addition, the study also suggests that several simultaneous stress factors may cause the metabolome to go back to an organisation that resembles the unstressed metabolome.

The paper by Wang et al. is an example of insights into developmental plant biology. Using the coniferous tree *Dacrydium pectinatum* as a study system, this paper focuses on detailing the hormonal dynamics during the developmental phases of cone creation. The paper demonstrates how bioinformatic analyses are a fast and reliable way to obtain insights into reproductive development, which will help in studying the challenges of low seed quality and poor natural regeneration.

The review by Yadav et al. treats several integrated omics techniques (genomics, transcriptomics, and metabolomics) to understand stress responses in flax and to determine a set of underlying regulatory mechanisms. Through the lenses of various omics technologies, this review guides us into an in-depth understanding of the genomic architecture, relevant signaling pathways, and physiological adaptability of flax under stress. Integrating omics technologies on several flax genotypes with significant trait variation, according to the review, will reveal previously unknown flaxseed variables, paving the way for stress-tolerant variant breeding. The study also concludes that significant omics techniques are less well represented in the literature, such as proteomics, phenomics, and ionomics.

Post-harvest analyses identify yet another use of omics techniques to meet modern challenges with food supply and storage. In the study by Dey et al., a comprehensive online resource is presented that provides the opportunity to search for genes that code for important traits and functions. The authors discuss the various long non-coding RNAs such as lncRNAs, circRNAs, and miRNAs and their role in the delayed ripening of diverse plants, including cucumber. The study thus provides an argument for the expression of a gene that may be used as a biomarker for postponed shelf life.

Genomic data allow us to locate genes with varied expression patterns, pointing at the fundamental core of biology’s central dogma. Because the genome is more stable than the transcriptome and metabolome, it has proven to be a strong way to identify genetic diversity. The molecular responses of plants during growth and development are ephemeral in their nature, and their functions and modes of action will affect the final product or phenotype. It is this gap between the genotype and the phenotype that the omics techniques may provide insights into, and while these techniques result in massive amounts of data, the bioinformatics analyses emerge as a new barrier that we must pass to enable our ability to predict the consequences of various biotech solutions.

## Author contributions

RG, DY and BRA wrote and edit the manuscript and approved it for further submission.

